# Deep Learning without Weight Symmetry

**Published:** 2024-05-31

**Authors:** Li Ji-An, Marcus K. Benna

**Affiliations:** Neurosciences Graduate Program, University of California, San Diego, La Jolla, CA 92093; Department of Neurobiology, University of California, San Diego, La Jolla, CA 92093

## Abstract

Backpropagation (BP), a foundational algorithm for training artificial neural networks, predominates in contemporary deep learning. Although highly successful, it is often considered biologically implausible. A significant limitation arises from the need for precise symmetry between connections in the backward and forward pathways to backpropagate gradient signals accurately, which is not observed in biological brains. Researchers have proposed several algorithms to alleviate this symmetry constraint, such as feedback alignment and direct feedback alignment. However, their divergence from backpropagation dynamics presents challenges, particularly in deeper networks and convolutional layers. Here we introduce the Product Feedback Alignment (PFA) algorithm. Our findings demonstrate that PFA closely approximates BP and achieves comparable performance in deep convolutional networks while avoiding explicit weight symmetry. Our results offer a novel solution to the longstanding weight symmetry problem, leading to more biologically plausible learning in deep convolutional networks compared to earlier methods.

## 1 Introduction

Both artificial and biological neural networks must orchestrate complex synaptic weight updates in order to improve task performance. The correct organization of these weight updates becomes even more challenging in deeper multilayer neural networks, often referred to as the credit assignment problem. Over the past decades, the error backpropagation (BP) algorithm has revolutionized contemporary deep learning [1], serving as a fundamental algorithm for training artificial neural networks.

Despite its success, BP is frequently considered biologically implausible. Although recent proposals have (partially) addressed many biological implausibilities, such as nonlocal plasticity, multiple separate learning phases, and non-biological error representations [2, 3, 4, 5], a significant limitation known as the weight symmetry (or weight transport) problem persists [1, 6, 7, 8]. In BP, following the chain rule, the feedback weights $W^{T}$ in the backward pass are precisely symmetric to the feedforward weights W in the forward pass in order to accurately transmit error signals that match the gradients of the cost function. To implement BP in the brain, the error signals should be locally available for the feedforward weights, which implies that if $w_{a\to b}$ is the feedforward weight from neuron a to neuron b, there should exist a symmetric feedback weight from neuron b to neuron a (such that $w_{b\to a}=w_{a\to b}$). Why is this symmetric weight pattern not observed in the biological brain? Indeed, in local cortical circuits, two connected neurons a and b are either unidirectionally linked, with a probability of 69% (a→b or b→a), or bidirectional connected, with a probability of 31% (a→b and b→a) [9]. Even for bidirectionally connected neurons, $w_{a\to b}$ and $w_{b\to a}$ are only modestly correlated (R≈0.36) [9]. These observations are in striking contrast to the perfect correlation (R=1) for symmetric weight connectivity assumed by BP. This discrepancy thus calls for alternative explanations involving algorithms that biological mechanisms can implement.

In an effort to eliminate the symmetry assumption, it was demonstrated that random fixed feedback weights B can transmit useful error signals to upstream layers, leading to a learning process whereby the feedforward weights W approximately align with the feedback weights, i.e., $W\propto B^{T}$ (feedback alignment, FA) [10]. However, FA struggles to match BP’s performance in more advanced network architectures and in more challenging tasks, including deeper networks, convolutional layers, and large-scale image datasets (e.g., CIFAR10, ImageNet) [11]. A variant of FA, direct feedback alignment (DFA) [12], transmits error signals directly from the output layer to each upstream (hidden) layer, but also suffers from severe performance loss compared to BP [11]. Another FA variant was proposed by meta-learning the plasticity rule of feedforward weights to improve FA for online learning and low-data regimes [13], but it still significantly underperforms relative to BP.

Other proposals have explored various methods for updating feedback weights. In sign-concordant feedback (SF) algorithms, the sign of feedforward weights is transported (i.e., copied) to the feedback weights, both at initialization and during training [14]. This approach has shown considerable improvement over FA, approaching BP’s performance in simple tasks (though still with a significant performance gap in more complex tasks like ImageNet) [15, 16, 17]. Nonetheless, it remains unclear whether a biologically plausible plasticity rule can effectively transport the sign of the synaptic weight from the forward path to the corresponding synaptic weight in the backward path. In the weight mirror (WM) algorithm [18], the feedback weights are updated to track the feedforward weights, by injecting random noise into neurons during multiple learning phases (one phase for each layer). WM can reach a performance similar to BP; however, the biological feasibility of layer-specific learning phases and “bias blocking” (setting bias to zero) during the mirror mode remains to be established. The phaseless alignment learning (PAL) algorithm eliminates the need for the additional mirror mode, but again deviates from BP’s dynamics, showing a significant performance gap compared to BP [19]. The Kollen-Pollack (KP) algorithm [18], which uses a feedback weight update symmetric to the feedforward weight update and includes weight decay, has been shown to closely approximate BP, achieving similar performance. However, even starting from asymmetric initializations, both WM and KP ultimately lead to a scenario in which feedforward and feedback weights are symmetric, a connectivity pattern not observed in the brain. In short, KP and WM can achieve a BP-level task performance, but converge to a configuration with almost exact weight symmetry. FA, SF, and PAL alleviate the weight symmetry issue (although their weight configurations are still more aligned than and arguably incompatible with the biological observations), but significantly sacrifice task performance. None of these algorithms manages to achieve a BP-level performance while completely avoiding explicit weight symmetry.

In this study, we propose the Product Feedback Alignment (PFA) algorithm, which closely approximates BP. It completely avoids *explicit* weight symmetry by relying on alignment between forward and *indirect* backward pathways, using an additional population of neurons. Specifically, the feedforward weights W align with the product of a pair of feedback weights R and B (such that $W\propto (RB{)}^{T}$). We show that PFA can achieve BP-level performance in deeper networks, convolutional layers, and more challenging datasets such as CIFAR10 and ImageNet. Further, PFA can outperform other algorithms with sparse feedback connections, an important biological constraint of the brain. Our results offer a novel solution to the longstanding weight symmetry problem, providing supportive evidence for the feasibility of implementing BP-like algorithms in the brain.

## 2 Product feedback alignment

Consider a fully-connected multilayer neural network with depth L, mapping the input $x_{0}$ to the output $x_{L}$. In the forward pass, the activation of layer l+1 (with $N_{l+1}$ neurons), denoted as $x_{l+1}$, is determined by

(1) $$x_{l+1}=\sigma \left(W_{l+1,l}x_{l}+b_{l+1}\right),$$

where σ is the activation function, $W_{l+1,l}$ is the $N_{l+1}\times N_{l}$ feedforward weight, and $b_{l+1}$ is the bias. For a training dataset consisting of data points $\left(x_{0},y\right)$, the set of parameters $W_{l+1,l}$ and $b_{l}$ are trained to minimize the loss function ℒ that measures the difference between layer L’s output $x_{L}$ and the target output y (e.g., the cross-entropy).

**Figure 1:**
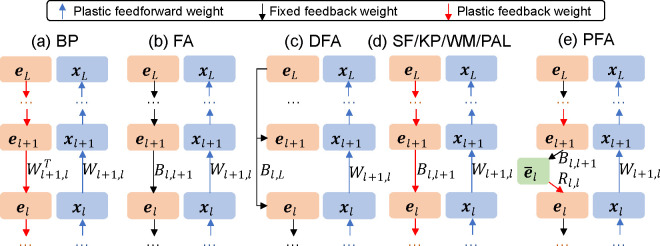
Comparison of learning algorithms for multilayer networks transmitting errors in the backward pass. BP: backpropagation. FA: feedback alignment. DFA: direct feedback alignment. SF: sign-concordant feedback. KP: Kollen-Pollack algorithm. WM: weight mirror. PAL: phaseless alignment learning. PFA: product feedback alignment.

The loss at the output layer directly provides the teaching signal (error) $e_{L}$ that is locally available at $x_{L}$, defined as the gradient $e_{L}=-\partial ℒ/\partial x_{L}$. In the backward pass of BP (Fig. 1), the error (i.e., gradient) at layer $x_{l}$ is iteratively backpropagated as

(2) $$e_{l}^{BP}=\sigma^{\prime }\left(x_{l}\right)⊙W_{l+1,l}^{T}e_{l+1}^{BP},$$

where ⊙ is the Hadamard product. Subsequently, the feedforward weight $W_{l+1,l}$ is updated by

(3) $$\Delta W_{l+1,l}=\eta e_{l+1}^{BP}x_{l}^{T},$$

where η is the learning rate. If we assume that $e_{l+1}^{i}$ (*i*-th component of $e_{l+1}$) is locally available at the neuron with activation $x_{l+1}^{i}$, then the update of $W_{l+1,l}^{i,j}$ (forward synaptic weight between $x_{l+1}^{i}$ and $x_{l}^{j}$) relies only on locally available information (the product of $e_{l+1}^{i}$ and $x_{l}^{j}$), which is biologically plausible. The aspect that lacks biological plausibility is the backpropagation of errors via $W_{l+1,l}^{T}$, since the same synaptic weight $W_{l+1,l}^{i,j}$ is used twice in both the forward and backward passes.

Because chemical synapses in the brain transmit information (carried by presynaptic spikes) only in one direction (from axon to dendrite), one must introduce an additional set of feedback weights $B_{l,l+1}$ to backpropagate the error signals from the output layer to upstream layers. FA employs fixed random feedback weights $B_{l,l+1}^{FA}\left(N_{l}\times N_{l+1}\right)$ that are independent of the feedforward weights, alleviating the weight symmetry problem. Then the error at the layer $x_{l}$ is computed as $e_{l}^{FA}=\sigma^{\prime }\left(x_{l}\right)⊙B_{l,l+1}^{FA}e_{l+1}^{FA}$. DFA similarly calculates the error at the layer $x_{l}$ using $e_{l}^{DFA}=\sigma^{\prime }\left(x_{l}\right)⊙B_{l,L}^{DFA}e_{L}$ via a fixed random feedback weight $B_{l,L}^{DFA}\left(N_{l}\times N_{L}\right)$. As shown in previous works, the feedforward weights $W_{l+1,l}$ in FA learn to gradually align with feedback weights $B_{l,l+1}^{T}$, leading to an approximate weight symmetry (to an extent that may still contradict biological observations). However, both FA and DFA failed to reach good performance for deeper networks, convolutional layers, and more challenging tasks. SF transports the sign of feedforward weights into backward weights, i.e., $B_{l,l+1}^{SF}=Sign\left(W_{l+1,l}^{T}\right)$, and the error $e_{l}$ is computed as $e_{l}^{SF}=\sigma^{\prime }\left(x_{l}\right)⊙B_{l,l+1}^{SF}e_{l+1}^{SF}$. SF significantly outperforms FA and DFA, but still underperforms compared to BP in deep convolutional layers for challenging tasks (e.g., ImageNet), and again exhibits approximate weight symmetry [17].

In PFA (Fig. 1), we introduce an intermediate population ${\overset{‾}{e}}_{l}$ (with ${\overset{‾}{N}}_{l}$ neurons) for each layer l, denoted as

(4) $${\overset{‾}{e}}_{l}=B_{l,l+1}^{PFA}e_{l+1}^{PFA},$$

using a fixed random feedback weights $B_{l,l+1}^{PFA}\left({\overset{‾}{N}}_{l}\times N_{l+1}\right)$. Subsequently, the PFA error for layer l (with activations $x_{l}$) is calculated as

(5) $$e_{l}^{PFA}=\sigma^{\prime }\left(x_{l}\right)⊙R_{l,l}{\overset{‾}{e}}_{l},$$

where $R_{l,l}$ represents a plastic feedback weight matrix ($N_{l}\times {\overset{‾}{N}}_{l}$). In addition to the feedforward weights $W_{l+1,l}$ updated by $\Delta W_{l+1,l}=\eta e_{l+1}x_{l}^{T}$, we also update $R_{l,l}$ as

(6) $$\Delta R_{l,l}=\eta x_{l}{\overset{-}{e}}_{l}^{T}.$$

Note that the update of the synaptic weight $R_{l,l}^{j,i}$ only relies on locally available information $x_{l}^{j}$ and ${\overset{‾}{e}}_{l}^{i}$. The backward path between layers l+1 and l consists of a pair of feedback weights $B_{l,l+1}$ (omitting superscripts from here on for simplicity) and $R_{l,l}$. Weight decay is applied for both $W_{l+1,l}$ and $R_{l,l}$, such that the influence of initializations gradually diminishes. We will provide an explanation for the PFA’s *implicit* alignment mechanism in Section 4. We note that when the weight decay and learning rate for $R_{l,l}$ are set to zero, PFA effectively implements FA. When $B_{l,l+1}$ is set to an identity matrix (with ${\overset{‾}{N}}_{l}=N_{l+1}$), PFA reduces to the standard KP algorithm.

## 3 PFA approximates BP in MNIST handwritten digit classification

**Figure 2:**
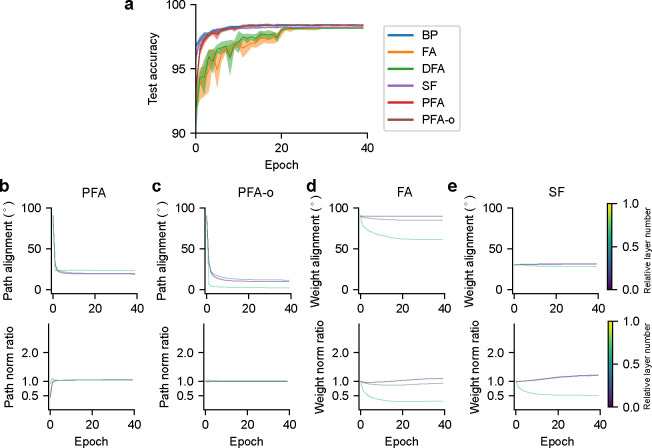
Characterization of learning algorithms for two-hidden-layer feedforward networks trained to classify MNIST digit images. (a) Task performance. Shaded regions show standard deviations across 5 seeds. PFA and PFA-o curves are almost overlapping with the BP curve, suggesting a close approximation. (b-e) Backward-forward weight alignment for FA/SF, and path alignment for PFA/PFA-o (top). Backward-forward weight norm ratio for FA/SF, and path norm ratio for PFA/PFA-o (bottom).

We used the above learning algorithms to train a two-hidden-layer feedforward network (with layer sizes 784–512-512–10) with ReLU activation on the MNIST dataset (using the BioTorch framework ^1^ [17]). The expansion ratio $\left(1/\lambda ={\overset{‾}{N}}_{l}/N_{l+1}\right)$ in PFA is set to 10. See Appendix A for training details. We found that PFA, PFA-o (a variant of the algorithm in which $B_{l,l+1}$ is chosen to be a semi-orthogonal matrix), and SF reach a test performance similar to BP, slightly outperforming FA and DFA (Fig. 2a).

We recorded several metrics throughout the training process. The first metric, backward-forward *weight alignment* [17], quantifies the angle between the feedforward weights and the corresponding feedback weights for FA, derived from the normalized inner products $\left(Vec\left(W_{l+1,l}^{T}\right),Vec\left(B_{l,l+1}\right)\right)$. We similarly define the backward-forward *path alignment* $\left(Vec\left(W_{l+1,l}^{T}\right),Vec\left(R_{l,l}B_{l,l+1}\right)\right)$ for PFA. Our PFA achieved an angle around 18° (Fig. 2b) for all layers in the network after the initial epochs (because we set a large initial weight decay for R and W). Due to the orthogonal initialization for B (Fig. 2c), PFA-o gradually achieved an angle close to 0°. These are substantially lower than the weight alignment angles for FA (between 60° and 90°; Fig. 2d) and SF (around 30°; Fig. 2e), indicating a superior alignment in PFA (but crucially without weight symmetry).

The second metric, backward-forward *weight norm ratio*, is designed to capture the risk of experiencing gradient exploding/vanishing problems observed in FA [16, 17], and is defined as ${∥B_{l,l+1}∥}_{2}/{∥W_{l+1,l}^{T}∥}_{2}$. We similarly defined the backward-forward *path norm ratio* ${∥R_{l,l}B_{l,l+1}∥}_{2}/{∥W_{l+1,l}^{T}∥}_{2}$ for PFA. Our PFA and PFA-o attained a weight ratio near 1 for all layers in the network – the ideal ratio akin to BP – after the initial epochs, suggesting better stability in error propagation over FA (between 0.3 and 1.1) and SF (between 0.5 and 1.2) (Fig. 2b–e). Collectively, these findings imply that PFA achieves a close approximation to BP.

## 4 Alignment mechanism between forward and backward paths in PFA

We now elucidate the alignment mechanism between the forward and backward pathways. During training, the feedforward weights $W_{l+1,l}$ align with the feedback weight matrix product ${\left(R_{l,l}B_{l,l+1}\right)}^{T}$ when the following two requirements are satisfied.

(1) $B_{l,l+1}^{T}B_{l,l+1}$ approximates an identity matrix. Then the weight updates for $W_{l+1,l}$ and ${\left(R_{l,l}B_{l,l+1}\right)}^{T}$ are aligned:

(7) $$\begin{array}{c} \Delta {\left(R_{l,l}B_{l,l+1}\right)}^{T}\;=B_{l,l+1}^{T}\Delta R_{l,l}^{T}=\eta B_{l,l+1}^{T}{\overset{-}{e}}_{l}x_{l}^{T}=\eta \left(B_{l,l+1}^{T}B_{l,l+1}\right)e_{l+1}x_{l}^{T}\\ \;\approx \eta e_{l+1}x_{l}^{T}=\Delta W_{l+1,l}\;. \end{array}$$

(2) The influence of the initial values of $W_{l+1,l}$ and $R_{l,l}$, which causes misalignment between $W_{l+1,l}$ and ${\left(R_{l,l}B_{l,l+1}\right)}^{T}$, further diminishes over successive learning epochs (due to weight decay).

Different approaches can ensure that $B_{l,l+1}^{T}B_{l,l+1}$ approximates an identity matrix. One approach is to initialize $B_{l,l+1}$ as a semi-orthogonal $\left({\overset{‾}{N}}_{l}\times N_{l+1}\right)$ matrix (with ${\overset{‾}{N}}_{l}\geq N_{l+1}$), as used in PFA-o.

**Figure 3:**
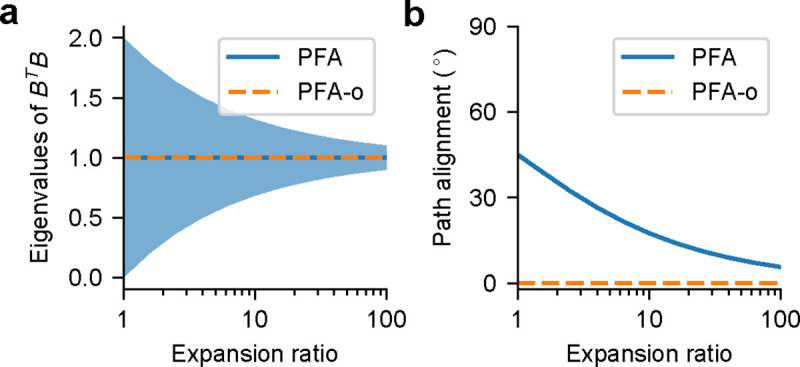
(a) Eigenvalues of $B^{T}B$ as a function of the expansion ratio (1/λ). The shaded region shows the standard deviation of the eigenvalues. (b) Backward-forward path alignment between W and ${\left(RB\right)}^{T}$ as a function of the expansion ratio (1/λ), where we randomly sampled W and set $R^{T}=BW$ (expected to hold after the effect of the weight initialization has fully decayed). This simplification is consistent with the path alignment after training observed in simulations.

Alternatively, elements in $B_{l,l+1}$ can be sampled independently from a distribution with a mean of 0 and a variance of $1/{\overset{‾}{N}}_{l}$, as used in PFA. This initialization for PFA is easier to implement in the brain than PFA-o, as it is not obvious what biological plasticity rule can learn a precisely semi-orthogonal matrix. Following the Marchenko–Pastur law [20] and assuming the limit $N_{l+1}\to \infty$ and ${\overset{‾}{N}}_{l}\to \infty$ with the ratio $N_{l+1}/{\overset{‾}{N}}_{l}=\lambda <1$, the eigenvalue density μ(v) of $B_{l,l+1}^{T}B_{l,l+1}$ satisfies

(8) $$\mu (v)=\frac{1}{2\pi }\frac{\sqrt{\left(\lambda_{+}\;-\;v\right)\left(v\;-\;\lambda_{-}\right)}}{\lambda v}1_{v\in \left[\lambda_{-},\lambda_{+}\right]}$$

with $\lambda_{\pm }={\left(1\pm \sqrt{\lambda }\right)}^{2}$. In the limit λ→0, $B_{l,l+1}^{T}B_{l,l+1}$ converges to the identity matrix with probability 1. See Fig. 3a for a numerical verification.

In short, although $W_{l+1,l}$ does not align with $R_{l,l}$ or $B_{l,l+1}$ individually, we still have $W_{l+1,l}^{T}\approx R_{l,l}B_{l,l+1}$ (see Fig. 3b) and thus $W_{l+1,l}^{T}e_{l+1}\approx R_{l,l}B_{l,l+1}e_{l+1}$, signifying a good approximation to BP (in the limit of ${\overset{‾}{N}}_{i}\to \infty$ for PFA). Like FA and DFA, PFA obviates the need to transport the feedforward weight (or its sign, as in SF) at initialization or during training, but significantly outperforms FA and DFA. Like KP, PFA employs aligned weight updates for both forward and backward pathways. However, unlike KP and WM, PFA does not lead to *explicit* weight symmetry, i.e., forward and backward synapses between any pair of neurons do not share identical weights for PFA (in fact we can avoid bidirectionally connected pairs of neurons altogether). For a detailed comparison of algorithms, see Table 1.

## 5 PFA for convolutional layers

Similar to FA and DFA for deep convolutional networks [16], PFA extends seamlessly to convolutional layers. Consider a forward convolutional kernel $W_{l+1,l}\in R^{h\times w\times N_{l+1}\times N_{l}}$, where h is the kernel height, w is the kernel width, and $N_{l}$ is the number of channels in the *l*-th layer. The feature-map activation $x_{l+1}^{i}$ for the *i*-th channel at layer l+1 is calculated as

(9) $$x_{l+1}^{i}=\sigma (\sum_{j=1}^{N_{l}}W_{l+1,l}^{i,j}\;\text{*}\;x_{l}^{j}+b_{l+1}^{i}),$$

where $W_{l+1,l}^{i,j}$ is the h×w kernel and * denotes convolution. The BP error (i.e., gradient) in the backward pass for *j*-th channel at the *l*-th layer is iteratively calculated as

(10) $$e_{l}^{\text{BP},j}=\sigma^{\prime }\left(x_{l}\right)⊙\sum_{i=1}^{N_{l+1}}{\tilde{W}}_{l+1,l}^{i,j}\;\text{*}\;e_{l+1}^{\text{BP},i},$$

where ⊙ is the Hadamard product, and $\tilde{W}$ is the a rotation of W by 180° (flipped kernel) [21]. The FA error for *j*-th channel at layer l is calculated as $e_{l}^{FA,j}=\sigma^{\prime }\left(x_{l}\right)⊙\sum_{i=1}^{N_{l+1}}\;B_{l,l+1}^{FA,j,i}\;*\;e_{l+1}^{FA,i}$, where $B_{l,l+1}^{FA}\in R^{h\times w\times N_{l}\times N_{l+1}}$ is the backward kernel. For PFA, the intermediate error for the *k*-th intermediate channel at the *l*-th layer is

(11) $${\bar{e}}_{l}^{k}=\sum_{i=1}^{N_{l+1}}B_{l,l+1}^{k,i}\;\text{*}\;e_{l+1}^{\text{PFA},i},$$

where $B_{l,l+1}\in R^{1\times 1\times {\overset{‾}{N}}_{l}\times N_{l}}$. The PFA error for the *j*-th channel at layer *l* is calculated as

(12) $$e_{l}^{\text{PFA},j}=\sigma^{\prime }\left(x_{l}\right)⊙\sum_{k=1}^{{\bar{N}}_{l}}{\tilde{R}}_{l,l}^{j,k}\;\text{*}\;{\bar{e}}_{l}^{k},$$

where $R_{l,l}\in R^{h\times w\times N_{l}\times {\overset{‾}{N}}_{l}}$. The pair of tensors $B_{l,l+1}$ and $R_{l,l}$ accommodate different convolutional hyperparameters for $W_{l+1,l}$ (e.g., stride, padding, dilation, groups). The weight updates are calculated similarly as $\Delta W_{l+1,l}^{i,j}=\eta e_{l+1}^{i}\;*\;x_{l}^{j}$ (in BP and PFA) and $\Delta R_{l,l}^{j,k}=\eta x_{l}^{j}\;*\;{\overset{‾}{e}}_{l}^{k}$ (in PFA).

## 6 PFA closely approximates BP in deep convolutional networks

**Figure 4:**
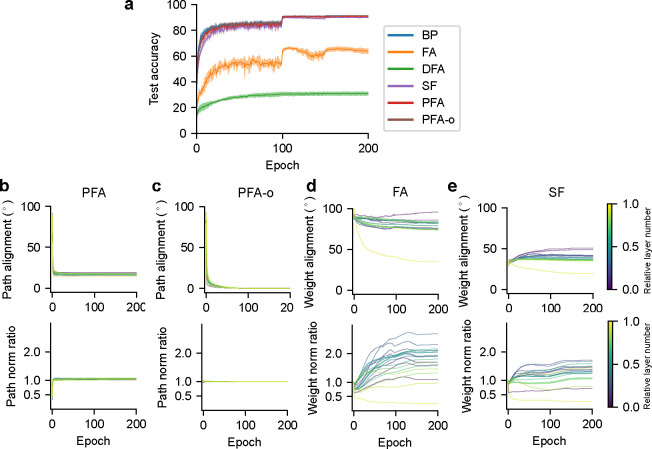
Characterization of learning algorithms for ResNet-20 on CIFAR-10. (a) Task performance. Shaded regions show standard deviations across 5 seeds. PFA and PFA-o curves are almost overlapping with the BP curve, suggesting a close approximation. (b-e) Backward-forward weight alignment for FA/SF, and path alignment for PFA/PFA-o (top). Backward-forward weight norm ratio for FA/SF, and path norm ratio for PFA/PFA-o (bottom).

We trained ResNet-20 on the CIFAR-10 dataset for these learning algorithms [22, 17]. See Appendix A for training details. Our results (Fig. 4a) show that networks trained with PFA and PFA-o attain a test accuracy comparable to that of BP and SF, significantly surpassing that of FA and DFA. PFA and PFA-o again achieve a close approximation to BP (Fig. 4b–e), indicated by the small angle of path alignment and the close-to-one backward-forward path norm ratio.

**Figure 5:**
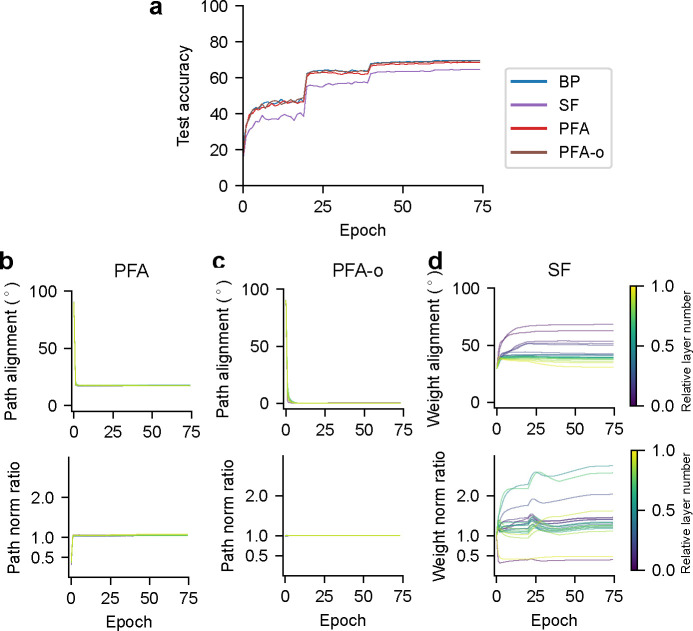
Characterization of learning algorithms for ResNet-18 on ImageNet. (a) Task performance. PFA and PFA-o curves are almost overlapping with the BP curve, suggesting a close approximation. (b-d) Backward-forward weight alignment for SF, and path alignment for PFA/PFA-o (top). Backward-forward weight norm ratio for SF, and path norm ratio for PFA/PFA-o (bottom).

We additionally trained ResNet-18 on ImageNet for these algorithms (except FA and DFA, which were previously shown to perform poorly [18, 17]). See Appendix A for training details. Our results (Fig. 5a) again show that networks trained with PFA (68.46%) and PFA-o (69.30%) achieve a test accuracy comparable to that of BP (69.69%), in a situation where even SF (64.63%) significantly underperforms. PFA and PFA-o again achieve a close approximation to BP (Fig. 5b–d), as shown by their path alignment and path norm ratio.

## 7 PFA for sparsely connected populations

**Figure 6:**
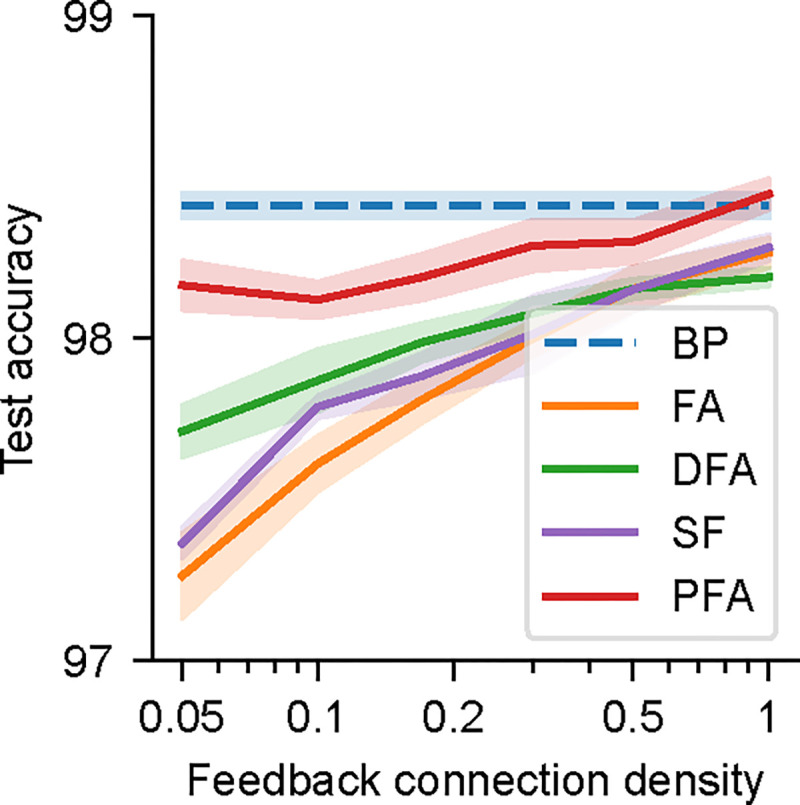
Effects of sparse connections in the feedback pathway, showing task performance on MNIST. A proportion of the feedback weights in FA, DFA, SF, and PFA (but not in BP) are fixed to zero during training. Shaded regions show standard deviations across 5 seeds.

In the biological brain, connections between neurons are sparse: in local cortical circuits, a large proportion of pairs of neurons are not connected [9], and the connection density decreases quickly over distance (between two neurons) [23]. The sparse connectivity is even more prominent in long-range projections. Therefore, biologically plausible rules should be able to overcome challenges caused by sparsity (or even take advantage of it). We thus examined the effects of different sparsity levels in the backward path on task performance, by fixing a proportion of feedback weights to zero during training (i.e., B in FA, DFA, SF, PFA, and also R in PFA). We found that the performance of FA, DFA, and SF gradually degrades with sparser connectivity (Fig. 6). In contrast, the performance of PFA degrades more slowly, suggesting a potential advantage of our PFA algorithm.

## Discussion

In conclusion, our PFA algorithm can approximate BP by fostering implicit alignment between forward and backward pathways, rather than relying on explicit weight symmetry. This algorithm provides a potential explanation for why we do not observe symmetric weights in the brain even though it presumably implements credit assignment across multilayer network architectures, serving as a demonstration of feasibility. We showed that PFA achieves a performance comparable to BP in both fully-connected feedforward networks and deeper convolutional networks, even on rather challenging datasets/tasks. In addition, our PFA outperforms other biology-inspired algorithms in a sparse-connectivity scenario.

Our PFA algorithm utilizes an additional neuronal population to transmit errors ${\overset{‾}{e}}_{l}$ to the population with activation $x_{l}$ within the same layer. This is broadly consistent with the brain’s diverse neuronal population structure, with predominantly local connections. We have demonstrated that introducing this population allows us to fully remove all bidirectional connections between neurons, leading to a weight alignment angle of 90° (i.e., zero weight correlation). To accurately match the weight correlation patterns in the brain, the observed values of weight alignment/correlation (an alignment angle of 78° and a correlation of R≈0.2, estimated in Appendix B) can be directly achieved by combining different groups of synapses separately learned by PFA (no correlation), FA (partial correlation), and KP (full correlation). Further, additional populations like the one introduced here provide extra flexibility and offer the possibility to deal with other biological constraints (such as sparse connections), a direction yet to be fully explored.

In our simulations, we fixed the feedback weights B, and since in PFA (but not PFA-o) each synaptic weight in B was independently sampled, a fairly large expansion ratio was necessary to ensure a close approximation to BP. Exploring biologically plausible plasticity rules for B that could reduce the expansion ratio requirement, potentially leading to a $B^{T}B$ closer to the identity matrix, is one of our future research directions. We only considered an expansion ratio larger than one (λ<1) in this study because an expansion ratio smaller than one will reduce the rank of the transmitted errors, similar to the scenario of low-rank gradient approximation [24]. Further investigation is required to understand the effects of low-rank weight updates.

The plasticity rules for updating the feedforward weights W, which are based on presynaptic activations and postsynaptic errors, are only biologically plausible when the error signal $e_{l}^{i}$ is locally available at the corresponding neuron with activation $x_{l}^{i}$. Several proposals have been put forward regarding how a single neuron can represent and transmit both forward activations and backward errors without interference. The dendritic cortical microcircuit framework posits that the errors are represented in the apical dendrites while the forward activations are represented in the basal dendrites [3]. An additional self-prediction pathway is introduced to facilitate error transmission. Another study, based on burst-dependent plasticity, proposes that the forward activations are represented by the event firing rates, while errors are encoded by burst probabilities [4], enabling the multiplexing of signals within the same neuron. Our PFA algorithm can be directly integrated with these proposals, providing solutions to the explicit weight symmetry problem in these frameworks.

Our study is subject to several limitations. First, while we solved the weight symmetry problem in feedforward networks, the convolutional kernels re-introduce weight sharing across spatial locations, potentially conflicting with biological observations again. Second, introducing the additional neuronal population increases the training time and memory cost compared to BP. Third, we only studied the effects of sparse feedback connections in MNIST, while more tasks/datasets are required to systematically examine them. Addressing these limitations is one of our future directions.

## Figures and Tables

**Table 1:** Detailed comparison of algorithms.

	BP	FA	DFA	SF	KP	WM	PAL	PFA

No need to transport weight sign	✗	✓	✓	✗	✓	✓	✓	✓
No need to transport weight magnitude	✗	✓	✓	✓	✓	✓	✓	✓
No separate feedback weight learning phase	✓	✓	✓	✛	✓	✗	✓	✓
No explicit weight symmetry after training	✗	✽	✽	✽	✗	✗	✽	✓
Accurate approximation to BP (path alignment)	✓	✗	✗	✗	✓	✓	✗	✓
BP-level task performance	✓	✗	✗	✦	✓	✓	✦	✓

BP: backpropagation. FA: feedback alignment. DFA: direct feedback alignment. SF: sign-concordant feedback. KP: Kollen-Pollack algorithm. WM: weight mirror. PAL: phaseless alignment learning. PFA: product feedback alignment.

✛ : It is unclear how the feedback weights in SF can be learned in a biologically plausible way.

✽ : these algorithms reduce, but do not fully eliminate explicit weight symmetry.

✦ : These algorithms significantly outperform FA and DFA, but still underperform compared to BP in more challenging tasks (CIFAR10 for PAL and ImageNet for SF).

## Footnotes

1 https://github.com/jsalbert/biotorch (Apache-2.0 license)

## A Training details

The expansion ratio $\left(1/\lambda ={\overset{‾}{N}}_{l}/N_{l+1}\right)$ in PFA is set to 10. All weights in the networks are initialized with the Glorot initialization with unit gain factor [25], except for the B weights in PFA, which are determined using the Glorot initialization with gain factor $\sqrt{(1+\lambda )/2}$, ensuring that $B^{T}B$ approximates an identity matrix (or B is directly set equal to a semi-orthogonal matrix for the “PFA-o” variant). For optimization, we employed stochastic gradient descent with a momentum of 0.9.

### A.1 MNIST

The learning rate is 10^−2^ for BP, SF, PFA, FA and DFA, multiplied by a factor of 0.5 at the 20th and 30th epoch. The weight decay coefficient is 10^−4^ for W in BP, FA, DFA and SF. It is initially 0.03 for W and R in PFA, but reduced to 10^−4^ after the first epoch. The networks are trained for 40 epochs with a batch size of 64 and 5 seeds. Training each network takes ~ 10 minutes on a GeForce RTX 3090.

### A.2 CIFAR10

The learning rate is 0.1 for BP, FA, SF and PFA, and 10^−3^ for DFA, multiplied by a factor of 0.1 at the 100th, 150th and 200th epochs. The weight decay is 10^−4^ for W in BP, FA, DFA and SF, and 0.005 for W and R in PFA (reduced to 10^−4^ after the first epoch). We trained the networks for 200 epochs with a batch size of 128 and 5 seeds. Training each network takes ~ 2 hours on a GeForce RTX 3090.

### A.3 ImageNet

The learning rate is 0.1 for BP, SF and PFA, multiplied by a factor of 0.1 at the 20th, 40th and 60th epochs. The weight decay is 10^−4^ for W in BP and SF, and 0.0005 for W and R in PFA (reduced to 10^−4^ after the first epoch). We trained the networks for 75 epochs with a batch size of 256. Training each network takes ~ 1 week on a GeForce RTX 3090.

## B Weight correlation/alignment in the brain

We ran simulations based on experimental observations [9]. We first sampled feedforward weights from a normal distribution. For 69% of feedforward weights (unidirectionally connected neurons), we set the corresponding feedback weights to 0. For the remaining 31% (bidirectionally connected neurons), we randomly sampled feedback weights that correlate with feedforward weights (correlation coefficient R=0.36). The weight correlation for all pairs of connected neurons is then R≈0.2, corresponding to a backward-forward weight alignment angle of 78°. This result is an approximate estimate and might change under different assumptions.
